# Concatenation, Conflict, and Complexity: Genealogical Heterogeneity Mimics Substitutional Heterogeneity for Nucleotide Model Selection

**DOI:** 10.1007/s00239-026-10317-4

**Published:** 2026-05-09

**Authors:** Jenniffer Roa Lozano, Mahamad Sayab Miya, Emma Turner, Duane McKenna, Richard Adams

**Affiliations:** 1 Department of Entomology and Plant Pathology, University of Arkansas, Fayetteville, AR 72701 USA; 2 Center for Agricultural Data Analytics, University of Arkansas, Fayetteville, AR 72701 USA; 3 Department of Biological Sciences, University of Memphis, Memphis, TN 38111 USA; 4 Center for Biodiversity Research, University of Memphis, Memphis, TN 38111 USA

**Keywords:** Akaike information criterion, Bayesian information criterion, Gene-tree discordance, Incomplete lineage sorting, Jukes–Cantor model, Multispecies coalescent model

## Abstract

Phylogenetic conflict—particularly hidden gene-tree discordance generated by incomplete lineage sorting (ILS)—is pervasive in multilocus and phylogenomic datasets, yet its consequences for nucleotide substitution model selection remain poorly understood. Modern molecular studies increasingly collect and concatenate large sets of independent loci sampled across distant and often poorly characterized regions of the genome, creating significant potential for heterogeneity when analyzed in combination. Here, we examine whether intra-alignment genealogical conflict can influence standard model selection procedures to favor parameter-rich substitution models even when sequences evolve under a simple substitution process. Through a series of in silico case studies, we simulated sequence evolution under the simplest rate-homogeneous Jukes–Cantor (JC69) model and generated concatenated alignments as mosaics of multiple loci, each evolving on its own gene tree drawn under the multispecies coalescent. Conflict was increased by manipulating conditions expected to elevate ILS and gene-tree heterogeneity and embedding progressively more hidden genealogies within alignments while holding total alignment length constant. Despite all data being generated under JC69, model selection frequently favored more complex models, with varying sensitivity depending on the number of taxa, the expected amount of conflict, and the specific selection criterion applied. A dominant pattern was frequent inclusion of among-site rate variation parameters (+ G4 and/or + I), and under extreme conflict, model selection increasingly favored richer substitution models (e.g., SYM, GTR). Broadly, our results showed that hidden conflict can manifest as substitutional and rate heterogeneity, driving selection procedures to compensate with additional parameters in concatenated analyses under high conflict. Broadly, our study contributes to a greater understanding and appreciation of the challenges in modeling molecular evolution in the era of multilocus phylogenetics.

**Supplementary Information:**

The online version contains supplementary material available at 10.1007/s00239-026-10317-4.

## Introduction

Few topics in modern evolutionary research have earned as much notoriety as phylogenetic conflict. Studies have documented, tested, refined, and debated the causes and consequences of conflict across numerous contexts, applications, data types, and scientific questions covering a range of taxonomic groups (Smith et al. 2020; Morales-Briones et al. 2021; Parins-Fukuchi et al. 2021; Hughes et al. 2023; Myers et al. 2024; Joyce et al. 2025). Collectively, this work has demonstrated that phylogenetic conflict is real and widespread across molecular and morphological data (Philippe et al. 2011; Smith et al. 2015; Keating et al. 2023), and it can have devastating impacts when ignored (Zhang et al. 2015; Parins-Fukuchi et al. 2021). While many studies have examined the impacts of unmitigated conflict on topology and branch length estimation (e.g., Wen and Nakhleh 2018; Blair and Ané 2020; Zhao et al. 2022), other aspects of evolutionary analyses can also be affected, such as species delimitation (Yang and Rannala 2010; Noguerales et al. 2018; Chan et al. 2023), selection analyses (Venkat et al. 2018), tests of trait coevolution (Cope et al. 2020; Parins-Fukuchi et al. 2021), and divergence time estimation (Carruthers et al. 2022; Höhna et al. 2025). Thus, it is widely accepted that phylogenetic conflict poses a significant impediment to our understanding of molecular biology and evolution (Jeffroy et al. 2006; Steenwyk et al. 2023; Adams et al. 2025).

Discordance between phylogenies can manifest in myriad ways and arise from biological and methodological sources (Dávalos et al. 2012; Smith et al. 2020, 2025). Conflict often occurs naturally from processes of speciation and diversification, notably including incomplete lineage sorting (ILS), horizontal gene transfer, natural selection, hybridization, introgression, and recombination (Adams et al. 2018; Hibbins and Hahn 2022; Sanderson et al. 2023; Zhang et al. 2024a; Shi and He 2025). ILS is arguably the most well-studied source of conflict that has been documented widely across the Tree of Life (Tan et al. 2023; Xie et al. 2024; Zhang et al. 2024b, 2025). ILS often occurs during rapid speciation events, when new lineages descend from ancestors within a short period, thereby allowing ancestral polymorphisms to persist even after descendant lineages have diverged (Maddison and Knowles 2006; Avise and Robinson 2008). Gene tree discordance can generate a phenomenon known as hemiplasy (Avise and Robinson 2008), which can be a concern for evolutionary inference (Copetti et al. 2017; Yan et al. 2022; Greenwood et al. 2025). Recent studies have shown that ignoring hemiplasy creates an illusion of homoplasy, leading to misleading evidence of multiple substitutions when analyzing loci comprising discordant trees (Robinson and Ropiquet 2011; Chira and Thomas 2016; Wu et al. 2018; Azevedo et al. 2022). Hemiplasy can generate false signals of heterogeneity in both rate and substitution patterns uncovered from molecular data (Hahn and Nakhleh 2016; Mendes and Hahn 2016; Guerrero and Hahn 2018). From a methodological perspective, the types of phylogenetic datasets and the methods used to create and analyze them can also contribute to conflict (Nater et al. 2015; Reddy et al. 2017; Simmons et al. 2022; Steenwyk et al. 2023).

The multispecies coalescent (MSC) model was developed to combat the negative effects of ILS by modeling the distribution of genealogies as a function of the species tree (Liu and Pearl 2007; Degnan and Rosenberg 2009; Heled and Drummond 2010). The MSC has been widely adopted through evolutionary research as a framework for viewing species trees from a population-genetic perspective, while accounting for the processes that ultimately influence genealogical variability across the genome (Maddison 1997; Flouri et al. 2018; Liu et al. 2019; Jiao et al. 2021; Kornai et al. 2024). MSC-based frameworks have since proved a robust foundation for species tree reconstruction, diversification analyses, trait modeling, and numerous other dimensions of multilocus phylogenetics (Rannala and Yang 2003; Zhong et al. 2013; Edwards et al. 2016; Yan et al. 2023).

Selection of a probabilistic model of nucleotide substitution is often considered a first step towards accurate phylogenetic inference (Posada and Crandall 2001; Hoff et al. 2016), though see recent arguments (Abadi et al. 2019; Tao et al. 2020; Fabreti and Höhna 2023). Popular nucleotide substitution models span the simple Jukes-Cantor (JC69) model (Jukes and Cantor 1969) to the more complex General Time Reversible (GTR) model (Tavaré 1986), alongside many models of varying complexity (Posada and Buckley 2004; Yang 2014; Arenas 2015). These models differ in the number of parameters and the types of substitution patterns and processes considered, including relative transition/transversion rates, equilibrium base frequencies, and whether rate variation among sites is included or not (Yang 1994; Sullivan and Swofford 2001; Arenas 2015). Given a specific alignment, the process of selecting a model typically involves the comparison of the relative fit and complexity of candidate models to identify the “best” model that most effectively describes processes influencing sequence evolution (Posada and Buckley 2004). Overly complex models include too many unnecessary parameters, while overly simple models fail to capture realistic features of evolution; both scenarios can be problematic (Sullivan and Swofford 2001). Therefore, model selection seeks to balance fit and complexity. In practice, information criteria techniques are commonly used, including notably Akaike Information Criterion (AIC) (Akaike 1974), corrected Akaike Information Criterion (AICc) (Hurvich and Tsai 1989), and Bayesian Information Criterion (BIC) (Schwarz 1978).

Previous research has hinted at potential risks of phylogenetic conflict on evolutionary model selection (Kubatko and Degnan 2007; Mendes and Hahn 2016; Springer and Gatesy 2016). Nucleotide substitution models are the de facto standards for studying molecular evolution and phylogenetic relationships, as they provide the likelihood function of observing nucleotide site patterns given a tree topology, set of branch lengths, and evolutionary parameters (Yang 2014). Hence, the model selection procedure influences estimates of tree topology and other phylogenetic components (Sullivan and Swofford 2001; Hoff et al. 2016), branch lengths (Buckley 2002), and other parameters (Lemmon and Moriarty 2004). Importantly, conventional approaches for model selection typically assume that all sites within an alignment share the same tree according to the canonical phylogenetic likelihood function. Yet, this key assumption rarely holds when exons, introns, or other loci are concatenated and analyzed together as a single combined alignment (Yang 1996; Gadagkar et al. 2005).

Here, we ask: can hidden gene tree conflict within concatenated alignments influence nucleotide substitution model selection procedures? We conducted a series of simulation experiments with escalating conflict to answer this question across a range of experimental and evolutionary conditions. Given previous findings that conflict can inflate estimates of substitution rates and patterns (Mendes and Hahn 2016; Springer and Gatesy 2016; Guerrero and Hahn 2018), we hypothesized that intra-alignment conflict may cause model selection to favor overly complex models for sequence data generated by a simple process. That is, we targeted the specific hypothesis that high levels of conflict can influence model selection procedures to favor parameter-rich models for simplistic data. To test this hypothesis, we simulated evolution for all sequences according to the simplest, rate-homogenous JC69 model for concatenated alignments that were constructed as a mosaic of multiple loci, each with its own associated gene tree. Holding the total alignment length constant, we varied the amount of conflict and discordance within alignments and conducted model selection using three popular information-criteria: BIC, AIC, and AICc. Our goal is not necessarily to determine whether these methods “fail” but instead we seek to better understand how they “behave and adapt” to conflicting signals when applied to concatenated alignments with hidden genealogical heterogeneity. In this context, selecting additional parameters (e.g., + G4 or + I) should be interpreted in context: such parameters suggest that models may provide a phenomenological fit to locus-to-locus genealogical and branch-length variation rather than indicate true among-site rate variation on a shared genealogy (see Discussion). While not exhaustive, our study seeks a new perspective on model selection under conditions of hidden conflict likely to be encountered when concatenating distinct multilocus datasets.

Methods

### A Simulation Case Study: Does Conflict Affect Model Selection?

**Fig. 1:**
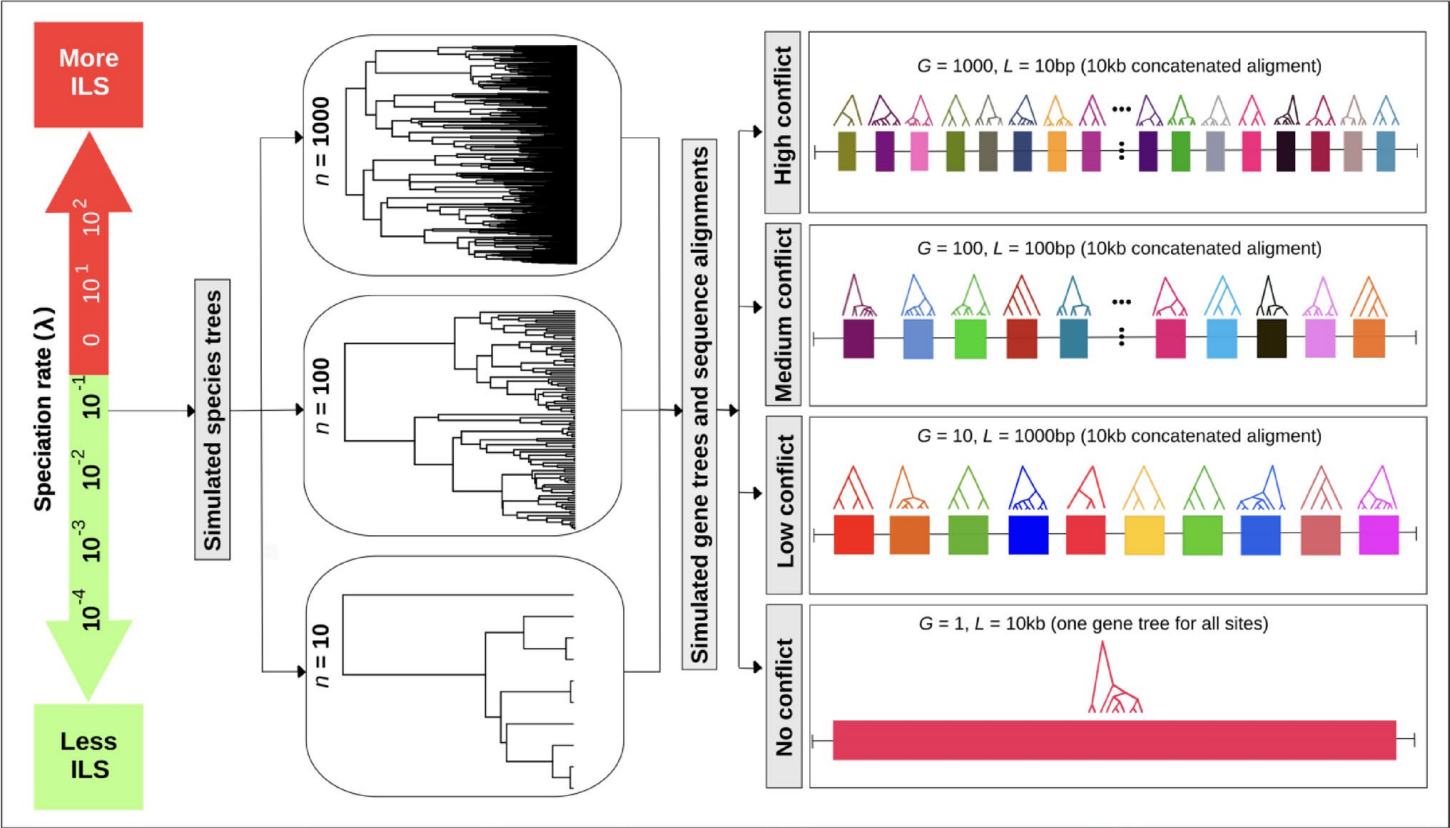
Schematic illustrating experimental design for simulation conditions with varying speciation rate (left), simulated species trees with different numbers of species (center), and gene trees (right)

We conducted a multifactorial study to investigate the impacts of hidden genealogical conflict on model selection procedures. For this study, we focused on a straightforward scenario: all sequence alignments were generated under the simplest rate-homogenous, 4-state JC69 model (Jukes and Cantor 1969) across escalating conditions of conflict. Current methods of conventional phylogenetic inference (Gadagkar et al. 2005; Kubatko and Degnan 2007; Philippe et al. 2011) explicitly assume that all sites within an alignment share the same tree; our simulations, therefore, violate this assumption by incorporating varying degrees of hidden genealogical variation within concatenated alignments. We used the multispecies coalescent (MSC) as a platform for generating conflict, and our simulation conditions are designed to mimic empirical studies in which researchers often concatenate loci with distinct genealogical histories (Warnow 2015; Edwards et al. 2016), such as concatenating genes, exons, introns, or other loci into a single overall alignment (Fig. 1).

Our overall simulation protocol mirrors similar studies (Fabreti and Höhna 2023; Adams et al. 2025) with the following steps: (1) a species tree is simulated according to a birth–death diversification process with speciation rate $\lambda$ and death rate $\mu =\lambda /2$ (Yule 1925), (2) a set of $G$ gene trees is simulated based on the MSC model using the species tree obtained from the first step, (3) for each of the $G$ gene trees, an alignment of length $L$ is simulated according to the JC69 model, (4) these $G$ individual subalignments are combined together into a single concatenated alignment with a total length $L\times G$, and (5) nucleotide model selection is performed using the concatenated alignment by comparing the fit of 28 canonical substitution models belonging to seven overall model families (Table 1). Because we employed the MSC in step two, our study therefore focused on ILS-driven discordance; other sources of conflict (e.g., introgression, recombination) as well as gene tree estimation error itself may produce different patterns (see Discussion).

**Table 1:** List of 28 nucleotide substitution models belonging to seven model families alongside their associated degrees of freedom (df) and descriptions

Family	Specific Model	df	Description
JC	JC	0	Equal substitution rates and equal base frequencies (Jukes and Cantor 1969)
	JC + I	1	Includes portion of invariant sites (+ I)
	JC + G4	1	Includes among-site variation with gamma model (+ G4)
	JC + I + G4	2	Includes portion of invariant sites and among-site variation (I + G4)
F81	F81	3	Equal exchangeability with unequal base frequencies (Felsenstein 1981)
	F81 + I	4	Includes portion of invariant sites
	F81 + G4	4	Includes among-site variation with gamma model
	F81 + I + G4	5	Includes portion of invariant sites and among-site variation
K2P	K2P	1	Unequal transition/transversion rates and equal base frequencies (Kimura 1980)
	K2P + I	2	Includes portion of invariant sites
	K2P + G4	2	Includes among-site variation with gamma model
	K2P + I + G4	3	Proportion of invariant sites and among-site variation
HKY	HKY	4	Unequal transition/transversion rates and base frequencies (Hasegawa et al. 1985)
	HKY + I	5	Includes portion of invariant sites
	HKY + G4	5	Includes among-site variation with gamma model
	HKY + I + G4	6	Includes portion of invariant sites and among-site variation
TIM	TIM	6	Transition model, AC = GT, AT = CG and unequal base frequencies (Tamura and Nei 1993)
	TIM + I	7	Includes portion of invariant sites
	TIM + G4	7	Includes among-site variation with gamma model
	TIM + I + G4	8	Proportion of invariant sites and among-site variation
SYM	SYM	5	Symmetric model with unequal rates but equal base frequencies (Zharkikh 1994)
	SYM + I	6	Includes portion of invariant sites
	SYM + G4	6	Includes among-site variation with gamma model
	SYM + I + G4	7	Proportion of invariant sites and among-site variation
GTR	GTR	8	General time reversible model (Tavaré 1986)
	GTR + I	9	Includes portion of invariant sites
	GTR + G4	9	Includes among-site variation with gamma model
	GTR + I + G4	10	Proportion of invariant sites and among-site variation

The first row is the JC69 model, which was used to generate all sequencing data in this study

Throughout our simulations, we varied both the number of species and the expected amount of conflict within alignments based on the number of hidden gene trees, the number of species, and the expected internal branch lengths. Specifically, we evaluated three different tree sizes representing the number of species $n\in \left\{10, 100, 1000\right\}$. Additionally, we varied the expected amount of ILS using seven different speciation rates $\lambda \in \left\{{10}^{-4},{{10}^{-3},{10}^{-2},{10}^{-1},{10}^{0},{10}^{1},10}^{2}\right\}$ for the birth–death model used in the first step described above. The speciation rate $\lambda$ is inversely related to the expected divergence time between speciation events (Yule 1925; Stadler et al. 2016) and therefore shapes the predicted amount of gene tree conflict. Lower $\lambda$ reflects longer internal branches and lower conflict (less ILS), while higher $\lambda$ generates more opportunity for conflict and ILS as a result of shorter interior branch lengths (Rivas-González et al. 2023) (also see Fig. 1). Species trees were simulated using the *sim.bd.taxa.age* function provided in TreeSim version 2.4 (Stadler 2011), with a total root age of ten in coalescent time units. For each simulated species tree, a set of $G$ gene trees were generated according to the MSC using a population size of two in coalescent units with the *sim.coal.phylo* function from Phybase version 1.4 (Liu and Yu 2010). To first provide context for our simulation conditions, we computed Robinson-Foulds distance (Robinson and Foulds 1981) between each simulated gene tree and its corresponding species tree, as well as the fraction of phylogenetic informative sites to visualize evolutionary variation conflict across our simulations, suggesting moderate-to-high conflict scenarios (Figs. S1 and S2).

For a given combination of simulation settings, sequence alignments were generated for each of the $G$ gene trees using the Seq-Gen program (Rambaut and Grassly 1997), according to the standard rate-homogeneous JC69 model with a molecular clock and per-base, population-scaled mutation rate of $\theta =4{N}_{e}\mu = 0.01$ applied to all sites for all alignments. We varied the amount of conflict within the concatenated alignments by increasing the number of gene trees $G$ and the per-locus alignment length $L$ in base pairs (bp) simulated on each gene tree, while holding the total alignment length constant at 10kb. Specifically, we used four different scenarios of within-alignment phylogenetic conflict: (1) 10kb alignments generated from a single shared gene tree shared across all sites (“No-conflict”), (2) 10kb alignments that concatenate $G=10$ gene trees each with $L=1000$ bp (“Low-conflict”), (3) 10kb alignments that concatenate $G=100$ gene trees each with $L=100$ bp (“Medium-conflict”), and (4) 10kb alignments that concatenate $G=1000$ gene trees each with $L=10$ bp (“High-conflict”) (Fig. 1). By standardizing to 10kb alignments across all simulations, we provide a model selection procedure with the same total input data, while ensuring our analyses remain tractable for a large number of simulation replicates.

For each set of simulation conditions, we generated 100 replicates and performed model selection on each replicate using the ModelFinder option in IQ-TREE2 (Kalyaanamoorthy et al. 2017; Minh et al. 2020). For each simulation replicate, we evaluated whether the JC69 model was correctly selected, or conversely, model selection favored a more complex model with additional parameters. For example, evidence of bias towards overly complex models is recovered when the K2P model (Kimura 1980) or a GTR model (Tavaré 1986) is selected because all alignments were generated under the JC69 model. Evidence of model selection effects can also be identified if, for example, JC69 + G4 (JC69 with gamma-distributed rates) was chosen because an extra parameter (+ G4) was inferred but not used in the true generating process (though see the *Discussion* on this interpretation). We considered 28 possible models from seven model families, commonly used in popular programs such as MrBayes (Huelsenbeck and Ronquist 2001; Ronquist et al. 2012), RAxML (Stamatakis 2014), and IQ-TREE (Minh et al. 2020). These seven model families included the JC69 (Jukes and Cantor 1969), F81 (Felsenstein 1981), K2P (Kimura 1980), HKY (Felsenstein 1981; Hasegawa et al. 1985), TIM (Tamura and Nei 1993), SYM (Zharkikh 1994), and GTR (Tavaré 1986), while also considering the proportion of invariant sites (“I” option), among-site variation with the gamma model (“G” option), and both invariant sites and among-site variation (“I + G” option) (Table 1).

ModelFinder compares candidate nucleotide substitution models and identifies the best-fit model using the most widely used information-theoretic criteria. We assessed model fit with the AIC, which favors models that balance goodness-of-fit and the number of parameters (Akaike 1974); AICc (Hurvich and Tsai 1989), which adds a correction for finite sample sizes; and BIC, which imposes a stronger penalty for model complexity and small sample sizes (Schwarz 1978). We summarized the results of these model selection criteria (BIC, AIC, and AICc) by computing the frequency of selected models and their associated degrees of freedom across each set of simulation conditions.

## Results

### Hidden Genealogical Conflict can drive the Selection of Overly Complex Models

**Fig. 2:**
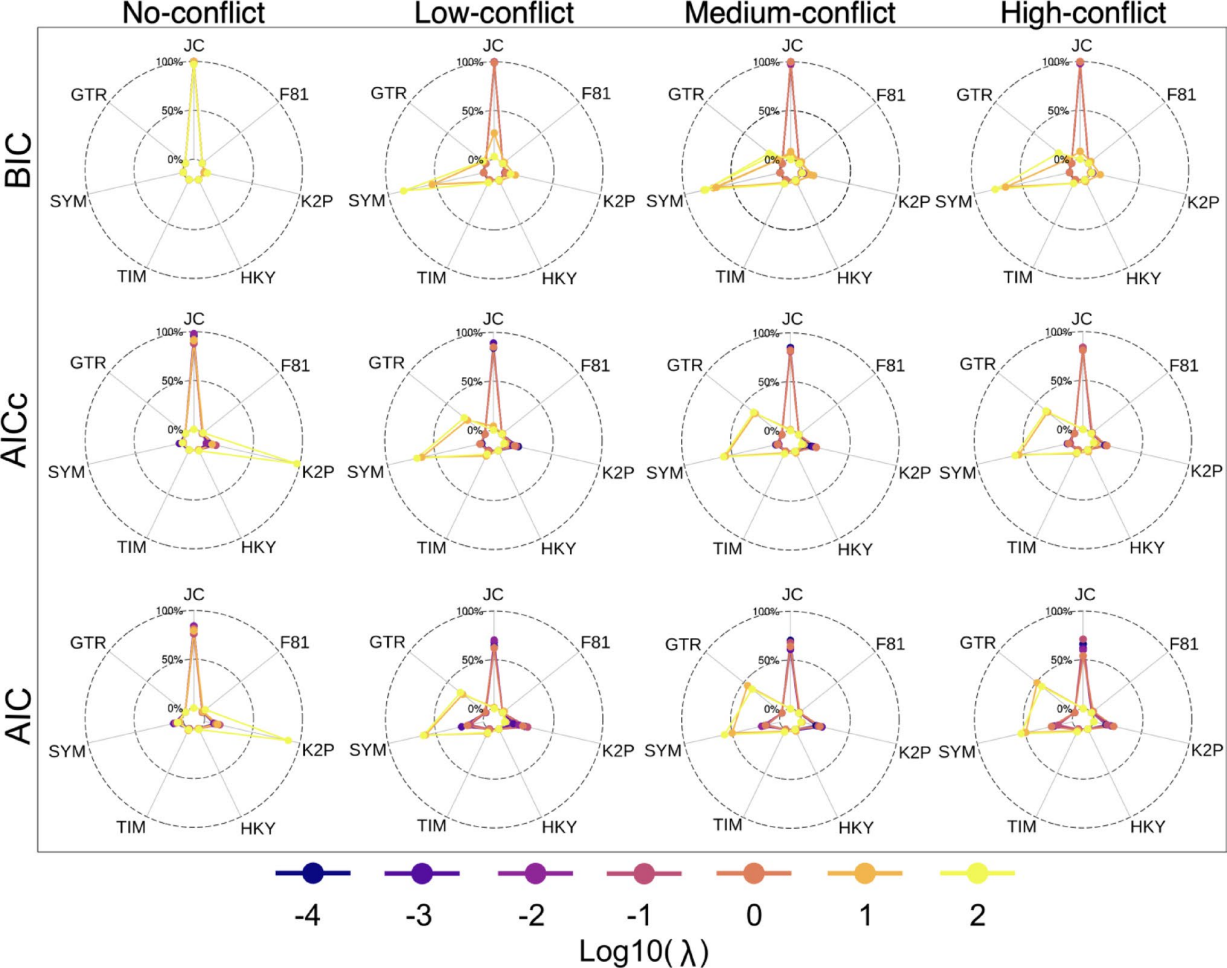
Radar plots showing selected model selection frequencies (0-100%) across four levels of conflict (columns), speciation rates (colors), and three model selection criteria (rows: BIC, AICc, and AIC) for analyses of large datasets with $n=1000$ species

**Fig. 3:**
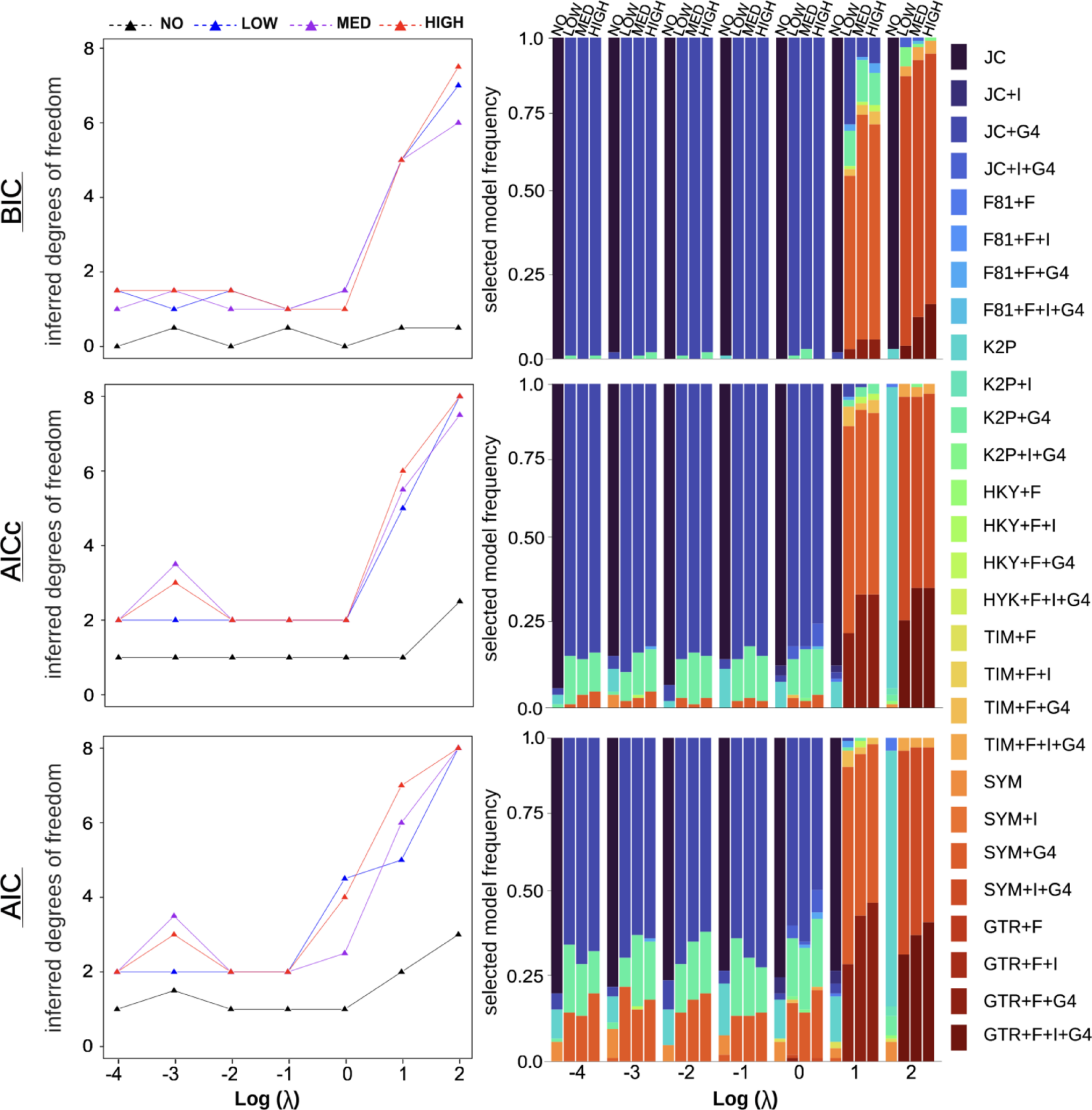
Results of nucleotide model selection across four levels of phylogenetic conflict and increasing speciation rates for analyses with $n=1000$ species. Panels in the left column show the average number of free parameters (degrees of freedom) inferred from model selection under each criterion (rows). Stacked bar plots in the right columns show the frequency fractions of the 28 substitution models selected for the conflict and speciation rate increase (left to right)

**Fig. 4:**
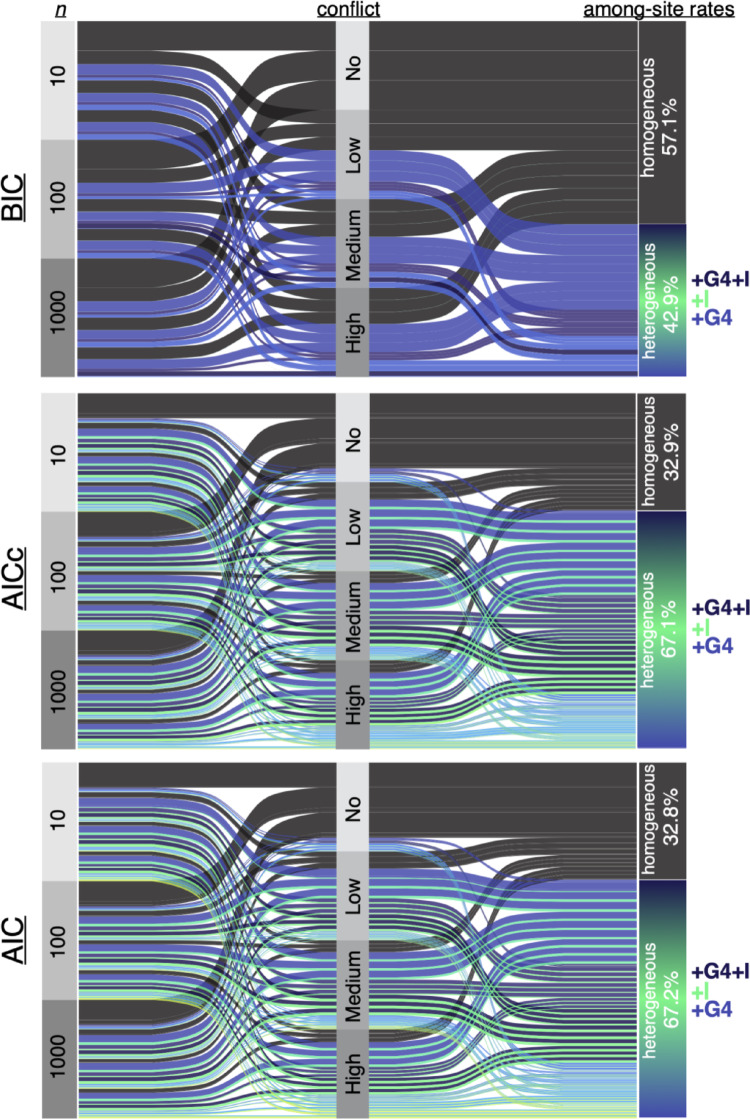
Alluvial plots dissecting the effects of conflict on estimates of among-site variation for BIC, AICc, and AIC. The plots track the fraction of replicates matching a given experimental configuration, with the number of species (left), the conflict level (center), and the resulting inference (right) for heterogeneous (+ G4, + I, + I + G4) versus homogeneous (equal rates) among-site rate models

Broadly, we find evidence that hidden conflict can influence model selection procedures for concatenated alignments to varying degrees (Figs. 2, 3, 4; S3-S6). Although all sequence data evolved according to the simple JC69 model, selection procedures sometimes favored overly complex substitution models depending on the conditions. A clear pattern emerged throughout our simulations: the effects of hidden conflict varied strongly as a function of the number of species $n$, the expected amount of ILS (proportional to $\lambda$), the total number of hidden gene trees $G$ embedded within alignments, and the specific criterion applied (BIC, AIC, or AICc). Because a single substitution model is fit to a mosaic alignment, shifts toward the selection of + G4/ + I and parameter-rich substitution families suggest that models are attempting to accommodate genealogical heterogeneity under misspecification, rather than as direct evidence that the underlying substitution pattern or process is complex itself.

The effects of hidden conflict are most evident when we examine the selected frequencies of the seven substitution model families for analyses of large phylogenies (Fig. 2). Under conditions of “No-conflict”, we find that BIC correctly selected the JC69 model with 100% accuracy regardless of the speciation rate. AIC and AICc also correctly selected JC69 at relatively high frequency with low speciation rates (~ 75—80%), yet both criteria showed reduced accuracy as they increasingly favored the K2P model with higher speciation rates (15—100%). As more conflict is added to the alignment (i.e., left to right), the three criteria increasingly selected complex models, particularly for high speciation rates (Fig. 2). For example, the SYM model was selected at frequencies of 72—80% for BIC, while AIC and AICc also selected SYM at frequencies > 50% when $\mathrm{log}\left(\lambda \right)$ > 0. Moreover, the GTR model was also selected at moderate frequencies (~ 24-51%) by both AIC and AICc. All three selection criteria were far less affected by conflict for analyses of smaller trees (Figs. S3 and S4). For example, the frequencies of K2P, SYM, and GTR decreased for trees when $n=100$ (Fig. S3), while the JC69 model was correctly selected at high frequencies (~ 65-100%) with only $n=10$ species, particularly for BIC (Fig. S4).

We find that these effects become clearer when dissecting the selected frequencies of the individual 28 models (seven families plus all four among-site heterogeneity options) across different simulation conditions (Figs. 3, S5, and S6). Again, selection of overly complex models was most readily apparent in analyses of large trees with $n=1000$ species (Fig. 3). Inferred degrees of freedom increased dramatically as the speciation rate and predicted conflict level increased for BIC, AIC, and AICc (left; Fig. 3). Reflecting this pattern, we find that all three criteria increasingly select models that include additional among-site heterogeneity parameters for the proportion of invariant sites (+ I), the gamma rates model (+ G4), or both (right; Fig. 3). For example, the JC + I, JC + G4, and JC + I + G4 models were increasingly selected when conflict is expectedly higher. AIC and AICc also selected the K2P, SYM, and GTR models with among-site heterogeneity parameters included, even with relatively low speciation rates, and BIC also selected heterogeneous SYM and GTR models when $\mathrm{log}10\left(\lambda \right)>0.$ Yet, in comparison with AIC and AICc, BIC appeared less sensitive to conflict when speciation rates are lower (top-to-bottom; Fig. 3). While broadly similar patterns were observed for analyses of smaller trees (Figs. S5 and S6), we find that model selection criteria tend to be more robust with higher frequencies of JC69, particularly when $n=10$. Yet evidence of bias towards increasingly complex models was still present, particularly when speciation rates were high and when $n=100$.

Lastly, we generated alluvial plots spanning the entirety of our simulated scenarios to parse our results based on the relative frequencies of selected models that either included parameters for among-site variation (“heterogeneous” with + I, + G4, or both) or did not include (“homogeneous”), regardless of the model class (Fig. 4). When comparing the three selection criteria, the largest fraction of rate-homogenous models was recovered with BIC across conditions. Specifically, BIC inferred homogeneous processes (i.e., no among-site variation) for 57.10% of all simulation replicates (top; Fig. 4). In contrast, both AIC and AICc exhibited a higher tendency to select models with rate heterogeneity across all conditions (center and bottom; Fig. 4). In the absence of conflict, all three information criteria tend to recover evolutionary models with rate homogeneity (“No-conflict”), yet higher conflict scenarios resulted in higher fractions of models that include among-site variation parameters (e.g., “High-conflict”; Fig. 4). As with our other results, we find that analyses of smaller trees (i.e., $n=10$) tended to also recover higher frequencies of rate homogeneous models, while larger analyses tend to be more susceptible overall.

## Discussion

Phylogenetic conflict is only expected to multiply in the age of whole genomes (Smith et al. 2015; Zhao et al. 2022; Steenwyk et al. 2023), and we are still learning about the challenges it poses for evolutionary inference. Previous studies have largely focused on the causes, consequences, and contexts of conflict in the process of phylogenetic reconstruction itself (Kubatko and Degnan 2007; Philippe et al. 2011; Springer and Gatesy 2016). Thus, our study contributes new insights into the effects of hidden conflict on model selection procedures for inferring the patterns and processes of molecular evolution from multilocus sequence data.

We find that the effects of conflict vary widely depending on the specific experimental and evolutionary settings. The clearest impacts were recovered specifically for analyses of large trees, as both the speciation rate and the number of gene trees embedded within alignments become elevated. These results are perhaps expected, as previous work has demonstrated that higher species sampling for analyses of large phylogenies increases the chance of ILS and gene tree discordance (Rokas et al. 2003; Maddison and Knowles 2006; Corl and Ellegren 2013). Likewise, higher speciation rates are expected to shorten the internal branches in the species tree, raising the likelihood of discordant genealogies embedded within concatenated alignments (Yule 1925; Stadler 2011; Rivas-González et al. 2023). ILS is often considered the most problematic source of genealogical conflict (Tan et al. 2023; Xie et al. 2024; Zhang et al. 2024b, 2025), and we find that such conflict can influence model selection under certain conditions. Yet, we find promising evidence that model selection can be relatively robust to conditions of low conflict, particularly for analyses of smaller datasets.

Concatenation remains a popular technique that seeks to increase the overall amount of phylogenetic information by combining distinct loci into a single alignment (Som 2015). However, this strategy can be problematic when joining distinct genes (Kubatko and Degnan 2007; Heled and Drummond 2010), the situation modeled in our simulation study. As a result, our findings suggest that these model selection criteria seek to explain hidden genealogical heterogeneity within alignments by selecting more complex models and additional parameters, a pattern previously observed in similar contexts (Luo et al. 2010; Susko and Roger 2020; Liu et al. 2023).

Our findings suggest a hierarchy of model complexity and error driven by hidden conflict. When conflict is low, model selection tends to favor models that more closely resemble the true data-generating process (i.e., the rate-homogeneous JC69). As conflict increases, however, more divergent and increasingly parameter-rich models (e.g., SYM, GTR + G4 + I) are selected with greater frequency. Across our simulations, a consistent pattern emerged: a preference for models that share the assumption of equal base frequencies, such as K2P and SYM. That is, information criterion sometimes favored more complex models with similar components, though in the extreme scenario, this was not always the case. Our supplementary analyses based on the HKY + G4 model revealed a similar, stepwise pattern: as conflict increased, models of progressively greater complexity than HKY were selected, whereas simpler models were never favored (see *Supplementary Materials*; Figs. S7-S9). Because many downstream analyses depend on specific modeling assumptions (e.g., unequal base frequencies or transition/transversion rate ratios), they are likely to be differentially influenced by these effects. For instance, some phylogenetic tree reconstruction methods are particularly sensitive to violations of base frequency assumptions (Fleming and Struck 2023; Zou et al. 2024), and approaches such as ancestral sequence reconstruction depend on the suitability of the assumed substitution model (Del Amparo and Arenas 2023). Future studies examining the downstream consequences of hidden conflict will therefore be essential to clarify its broader methodological implications across a broader range of evolutionary and experimental conditions.

Perhaps the most obvious symptom of hidden conflict was the tendency for criteria to adapt by selecting models that include extra parameters designed to capture among-site rate heterogeneity, such as the gamma rates model and proportion of invariant sites (or both). Though all sequence data were generated under the simplest JC69 model with a homogeneous rate shared across sites, the presence of intra-alignment conflict appeared to manifest as evidence of among-site variation. This observation makes sense, given that genealogies hidden within concatenated alignments likely differ not only in topology but in branch lengths as well, which likely contribute to signals of among-site variation. Thus, the observation that JC + G4 is often favored over the simpler JC can be interpreted as a tendency of models to accommodate conflicting signals within the alignment. From this perspective, the ability to compensate for conflict by adding among-site variation parameters may be argued as an advantage of model selection procedures. After all, the gamma-distributed rates model (+ G4) is only an approximation itself, which is not expected to be a perfect reflection of the true biological processes and causes of rate variation observed in real sequence data sampled from nature. Yet, under conditions of very high conflict, we still find evidence that information criteria increasingly favor parameter-rich models with complex substitution dynamics (e.g., SYM, GTR) in addition to among-site variation, likely because of the averaging of discordant signal (Philippe et al. 2011; Mendes and Hahn 2016; Parins-Fukuchi et al. 2021).

Notably, our results revealed consistent and interpretable differences among the three most used model selection criteria as observed when comparing BIC, AIC, and AICc. Among them, we find that BIC tended to be the most conservative and potentially less sensitive. For example, BIC often favored simpler models closer to the true JC69 model used to generate alignments, especially under scenarios of low to moderate levels of conflict and speciation rates. For high-conflict scenarios with low speciation rates, BIC often chose JC69 + G4, indicating only one additional parameter for among-site rate variation. However, for high conflict with larger trees and a higher speciation rate, BIC also started to favor more complex models, such as K2P + G4, SYM, and SYM + I + G4. This trend suggests that, although BIC may be more resilient to hidden conflict, it may still shift toward parameter-rich models as conflict intensifies and more taxa are included (Luo et al. 2010; Susko and Roger 2020; Liu et al. 2023). Previous studies have noted the more conservative behavior of BIC in other phylogenetic contexts (Abdo et al. 2005; Ripplinger and Sullivan 2008; Luo et al. 2010).

In contrast, AIC consistently showed a stronger tendency to overfit, favoring complex models even at relatively moderate conflict levels. This is reflected in the increased selection of models like K2P, HKY + F + I + G4, TIM + F + I + G4, and GTR + F + I + G4, at least for analyses of larger trees in high-conflict scenarios. AICc, designed to adjust for small sample sizes (Hurvich and Tsai 1989), exhibited a slightly more conservative tendency than AIC. However, AICc still often selected models with a substantial number of parameters, particularly K2P + G4, SYM + G4, and SYM + I + G4, as conflict increased for larger trees. Previous studies also found that AIC selects more complex models in phylogenetic analysis (Abdo et al. 2005; Ripplinger and Sullivan 2008). Our findings agree with previous arguments that selecting an appropriate selection criterion is as important as choosing a particular phylogenetic program or software (Li et al. 2025).

Our simulation case study raises the question: what are the empirical dangers of hidden phylogenetic conflict? Previous studies have demonstrated that such conflict significantly affects the estimation of phylogeny and other aspects of molecular evolution (Jeffroy et al. 2006; Steenwyk et al. 2023; Adams et al. 2025). If conflict is expected to be low, our results suggest that model selection procedures (particularly BIC) may be relatively robust. Moreover, the tendency for models to include among-site variation parameters may actually underscore the flexibility of these methods for adapting to real conflicting signals. In nature, phylogenetic conflict is thought to be widespread for a myriad of biological and technical reasons (Steenwyk et al. 2023). Predicting the amount of conflict and its underlying cause in a particular dataset remains a major challenge, and it can be difficult to determine how much variation within an alignment is due to incomplete lineage sorting, model misspecification, or other sources. Arguably, more work is needed to confidently answer this question across different evolutionary and experimental contexts.

Recent studies have argued for assuming the parameter-rich GTR + I + G model by default for phylogenetic inference, skipping the model selection step entirely (Abadi et al. 2019; Fabreti and Höhna 2023). Indeed, studies have suggested that overparameterization can be less of an issue than under parameterized models for certain types of tree reconstruction (Lemmon and Moriarty 2004). Yet, others have emphasized that model selection procedures can be used to identify the best-fit nucleotide substitution model rather than forcing the GTR model in order to improve topology and branch-length estimates (Hoff et al. 2016). Regardless, models of nucleotide substitution remain a bedrock of modern phylogenetics, not only for accurate tree inference but also for a deeper understanding of the patterns and processes of molecular evolution (Posada and Buckley 2004; Yang 2014; Arenas 2015; Fabreti and Höhna 2023). Therefore, we focused our study on the model selection process itself to provide insights into the potential effects of conflict on our ability to distinguish models of nucleotide substitution.

While our study provides new insight into these challenges, there are several limitations alongside new directions for future work. First, by simulating under the simplest JC69 model, we deliberately focused on the hypothesis that conflict will favor the selection of parameter-rich models for simple data (though see *Supplementary Materials* for a case study based on HKY). Extending this approach to other data-generating processes would likely help provide a broader picture of model selection and conflict. Second, we focused on ILS as the sole source of conflict, while there are many other potential drivers of conflict, such as horizontal gene transfer, natural selection, and hybridization (Hibbins and Hahn 2022; Keuler et al. 2022; Sanderson et al. 2023; Shi and He 2025). Recombination breaks up linked genealogies, which can bias both phylogenetic inference and sequence reconstruction when ignored (Schierup and Hein 2000; Arenas and Posada 2010). Alignment quality itself can also affect model selection as a source of error (Spielman and Miraglia 2021). Third, there are a number of other model selection frameworks beyond the information criterion approach studied here, including Bayes factors (Lartillot and Philippe 2004), decision theory (Minin et al. 2003), and hierarchical likelihood-ratio test (Frati et al. 1997), which may be complementary procedures for choosing evolutionary models. Yet, many current methods share the same standard phylogenetic likelihood function and its assumptions, suggesting that improvements to robustness may require novel strategies. Recent advances in machine learning also hold promise for improving robustness and accuracy of nucleotide model selection (Kulikov et al. 2024; Buch and Gambhava 2026), as do mixture modeling approaches (Pagel and Meade 2004; Bujaki et al. 2023; Gill et al. 2025). Understanding how these methods perform under conflicting conditions would be helpful, as each approach has its own advantages and limitations. Additionally, there is now a wealth of models proposed to study a diversity of evolutionary phenomena (see Arenas 2015; Ferreiro et al. 2026), representing targets for future investigations to expand beyond the 28 models examined here. Exploring new evolutionary and experimental conditions beyond the scope of our study would also help gain broader insights into conflict and model selection.

## Supplementary Information

Below is the link to the electronic supplementary material.

Supplementary Material 1
