# Supplementary material for: Concatenation, Conflict, and Complexity: Genealogical Heterogeneity Mimics Substitutional Heterogeneity for Nucleotide Model Selection
Source: J Mol Evol. 2026 May 9;94(3):527–40. doi: 10.1007/s00239-026-10317-4 (PMC13294338; doi:10.1007/s00239-026-10317-4)
Supplement: Supplementary file 1 — Supplementary Material 1 [file 239_2026_10317_MOESM1_ESM.pdf]

Supplementary Case Study: HKY+G4

To complement our investigations focused on simulations under the simplest JC69 model, we conducted supplementary analyses to evaluate model selection for data generated under the more complex HKY+G4 model. Here, sequence alignments were simulated following the same protocol described in the *Methods* section, with the only modification being the use of the HKY+G4 model (instead of JC69). Specifically, we simulated datasets using the HKY+G4 with a transition/transversion ratio of 4.0, equilibrium base frequencies of $f_A = 0.3, f_C = 0.2, f_G = 0.2$, and $f_T = 0.3$, and a value of 0.5 for the α parameter of the +G4 gamma rates model. All other aspects of the simulation procedure followed the strategy described in the main text, including simulations with $n = 10$, 100, and 1000 species across the same range of speciation rates and conflict conditions. For each set of conditions, we summarized the fraction of models selected across all replicates, with the HKY+G4 representing the true, correct generating process.

Our results show similar patterns to those uncovered in the primary analyses based on JC69 (e.g., comparing Fig. 3 versus Fig. S7). Generally, we find that increasing conflict can drive selection towards more complex models, depending on the information criteria as well as the specific evolutionary conditions (Figs. S7-S9). AIC appeared to be particularly susceptible to selecting the GTR family on trees with many species (Fig. S7), although these effects were less pronounced for smaller trees (Fig. S9). Across these simulations, model selection always selected models that were equally or more complex: the true, generating HKY+G4, as the JC69 model was never selected, for example. These results complement the primary analyses of the main text, illustrating a hierarchy of model selection following the baseline complexity of the true generating process.

SUPPLEMENTARY FIGURES

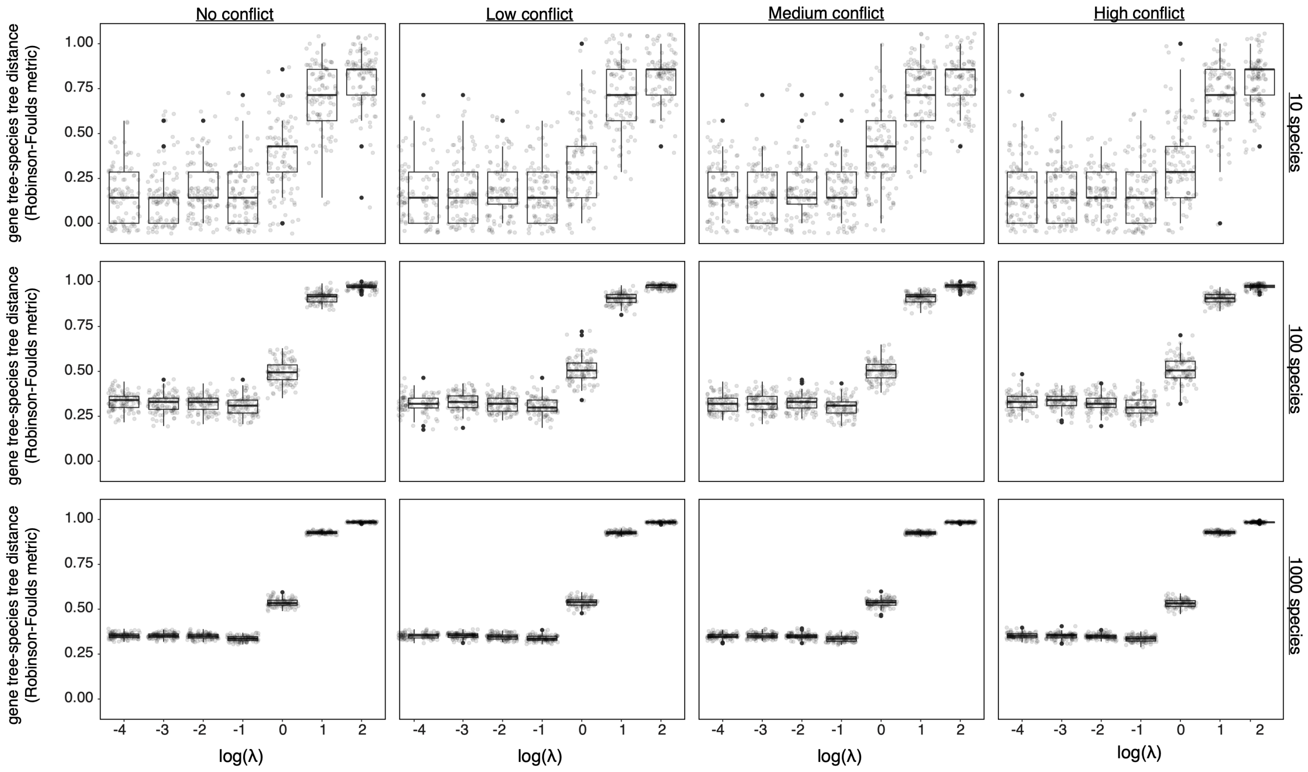

FIGURE S1. Boxplots showing distributions of gene tree-species tree distances across the evolutionary conditions explored in this study.

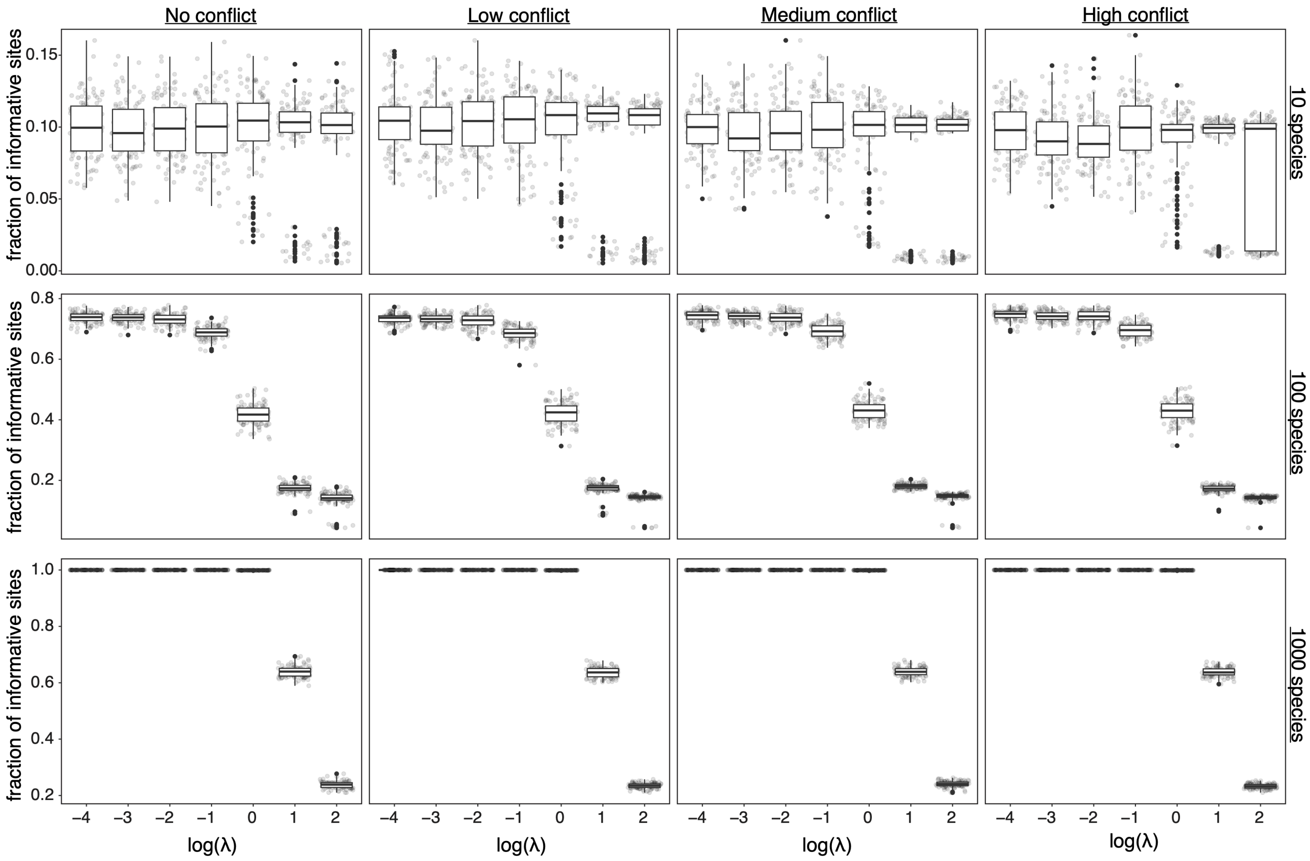

FIGURE S2. Boxplots showing distributions of the fraction of phylogenetic informative sites across the evolutionary conditions explored in this study.

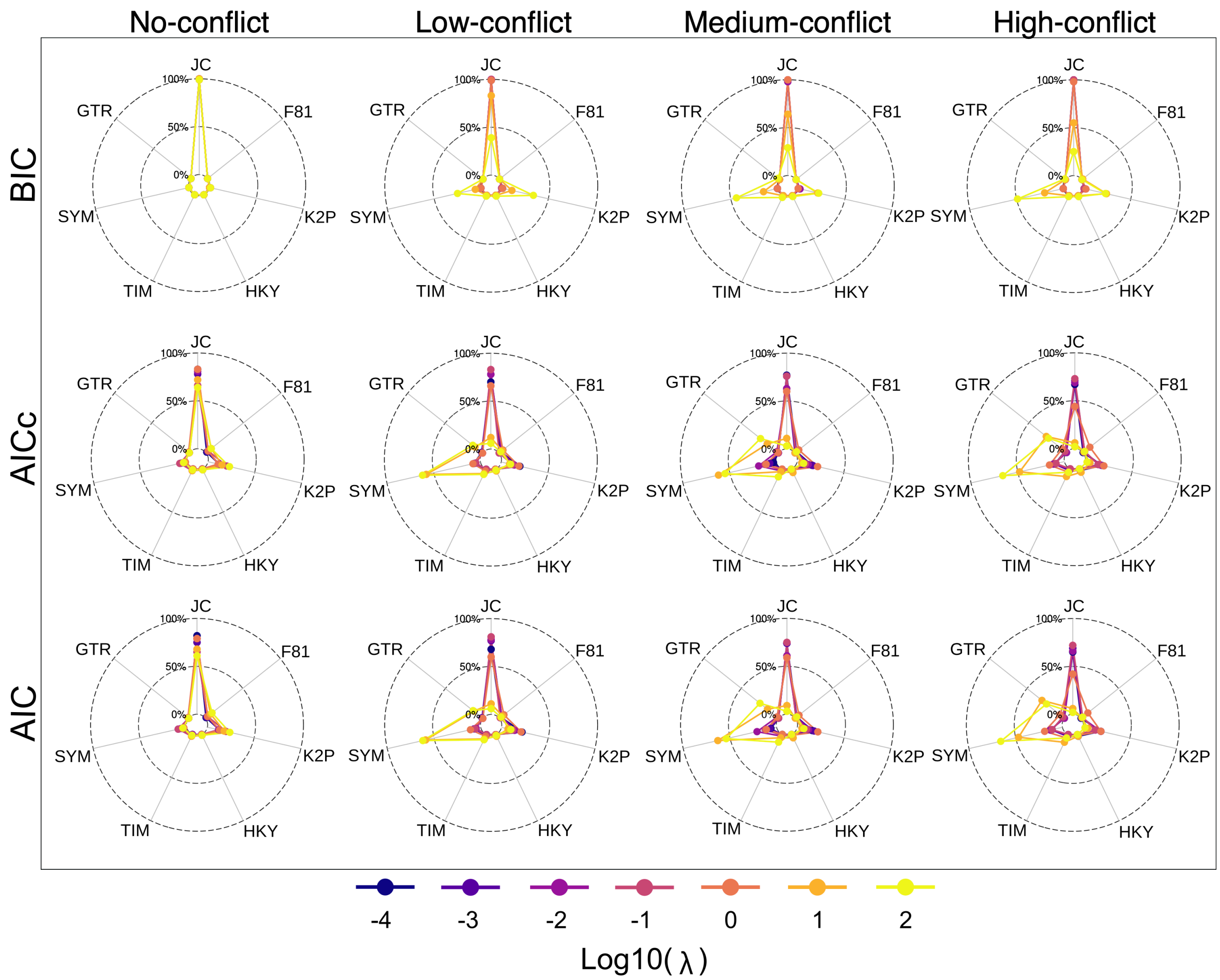

FIGURE S3. Radar plots showing selected model selection frequencies (0-100%) across four levels of conflict (columns), speciation rates (colors), and three model selection criteria (rows: BIC, AICc, and AIC) for analyses with $n = 100$ species.

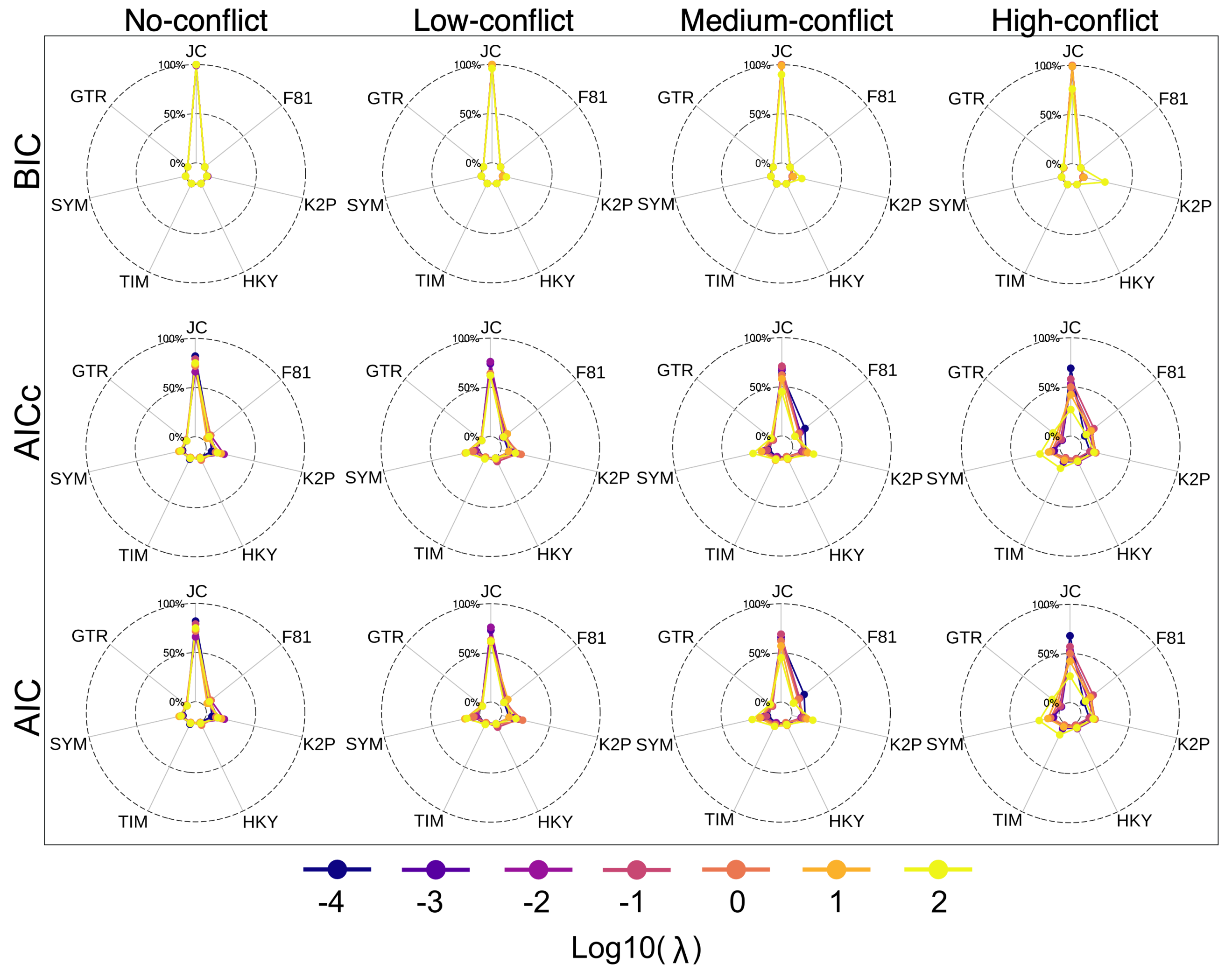

FIGURE S4. Radar plots showing selected model selection frequencies (0-100%) across four levels of conflict (columns), speciation rates (colors), and three model selection criteria (rows: BIC, AICc, and AIC) for analyses with $n = 10$ species.

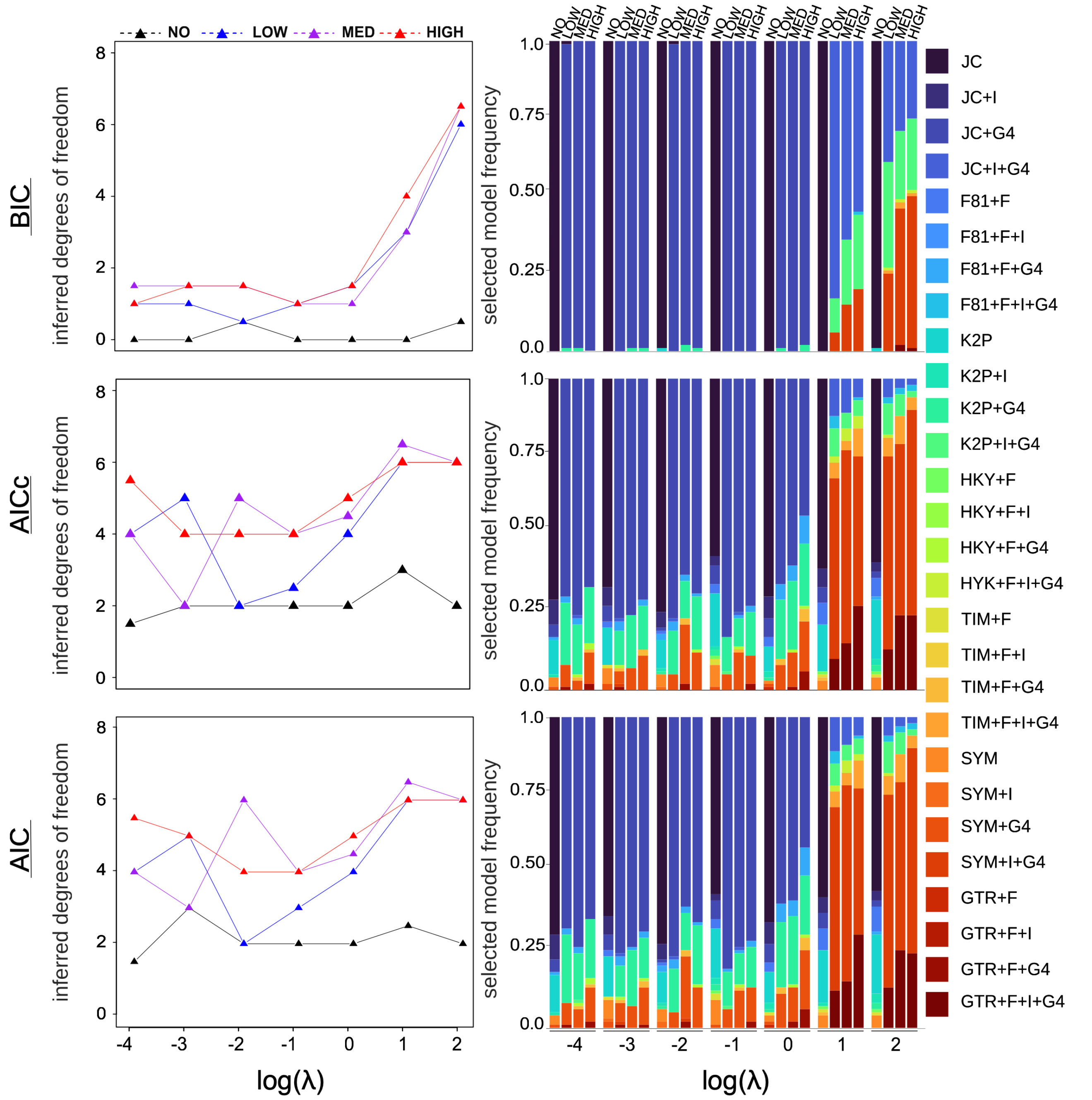

FIGURE S5. Results of nucleotide model selection across four levels of phylogenetic conflict and increasing speciation rates for analyses with $n = 100$ species. Panels in the left column show the average number of free parameters (degrees of freedom) inferred from model selection under each criterion (rows). Stacked bar plots in the right columns show the frequency fractions of the 28 substitution models selected for the conflict and speciation rate increase (left to right).

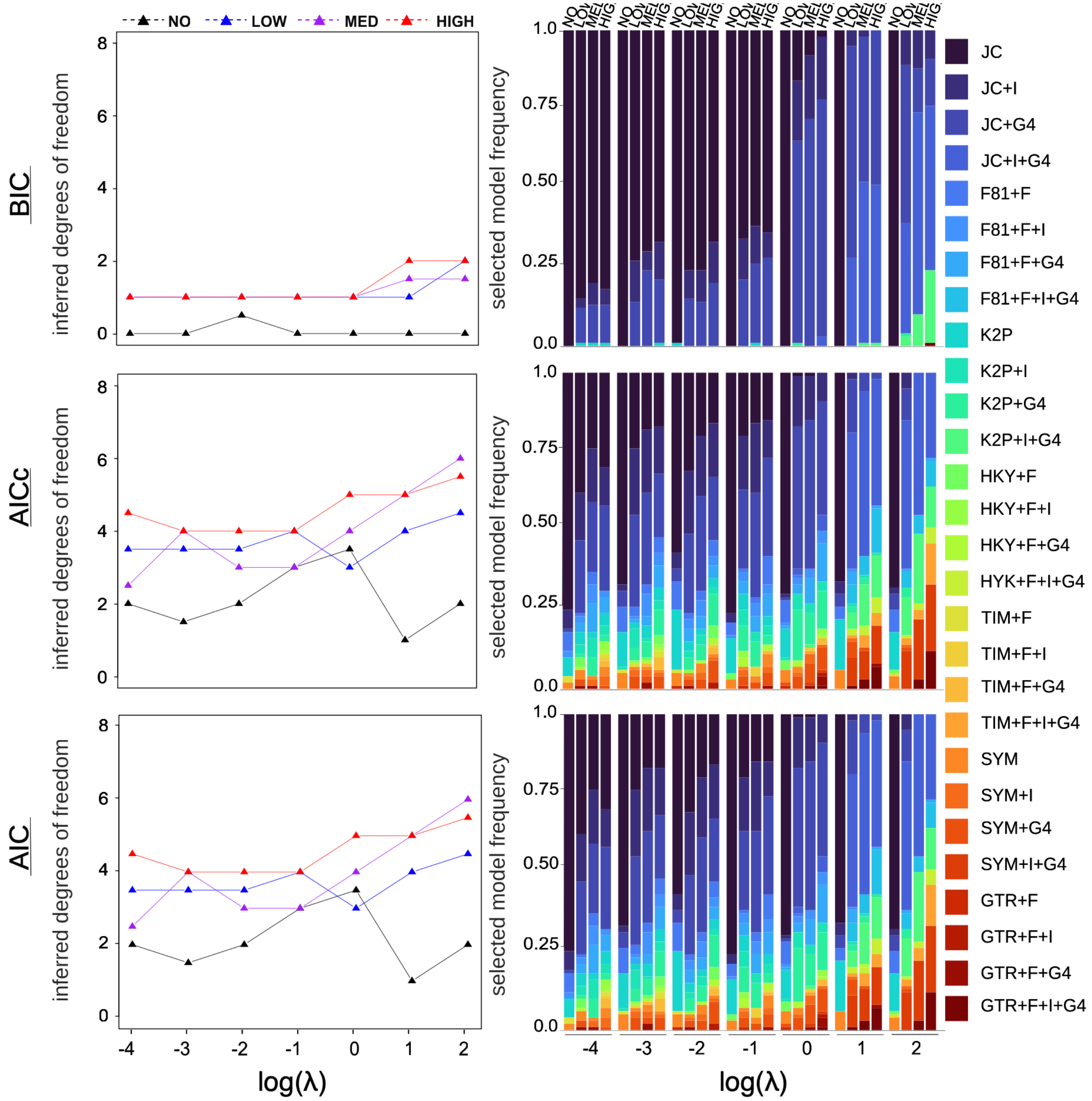

FIGURE S6. Results of nucleotide model selection across four levels of phylogenetic conflict and increasing speciation rates for analyses with $n = 10$ species. Panels in the left column show the average number of free parameters (degrees of freedom) inferred from model selection under each criterion (rows). Stacked bar plots in the right columns show the frequency fractions of the 28 substitution models selected for the conflict and speciation rate increase (left to right).

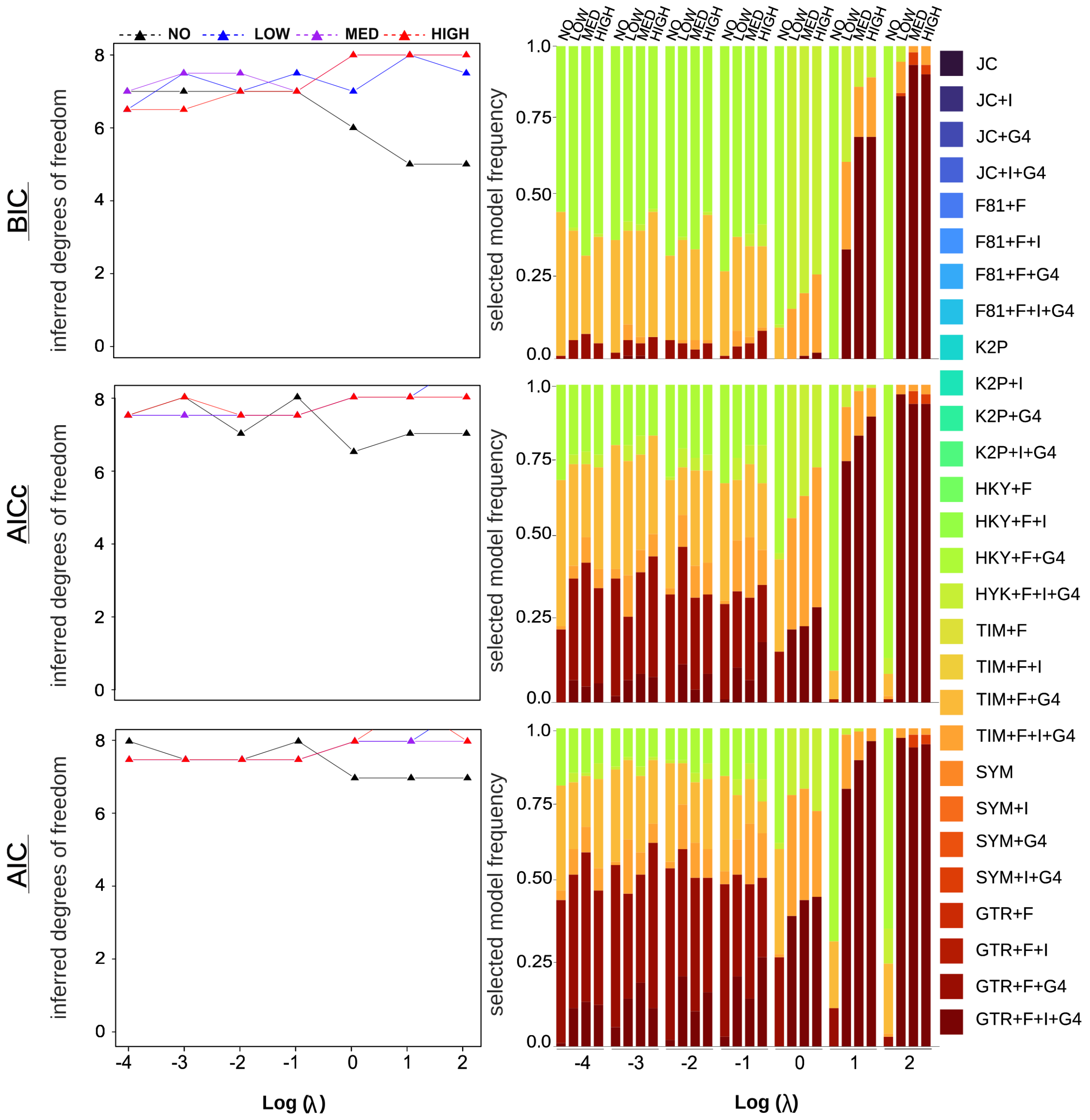

FIGURE S7. Results of nucleotide model selection across four levels of phylogenetic conflict and increasing speciation rates for analyses with $n = 1000$ species. Panels in the left column show the average number of free parameters (degrees of freedom) inferred from model selection under each criterion (rows). Stacked bar plots in the right columns show the frequency fractions of the 28 substitution models selected for the conflict and speciation rate increase (left to right). In these supplementary analyses, all data were generated according to the HKY+G4 model.

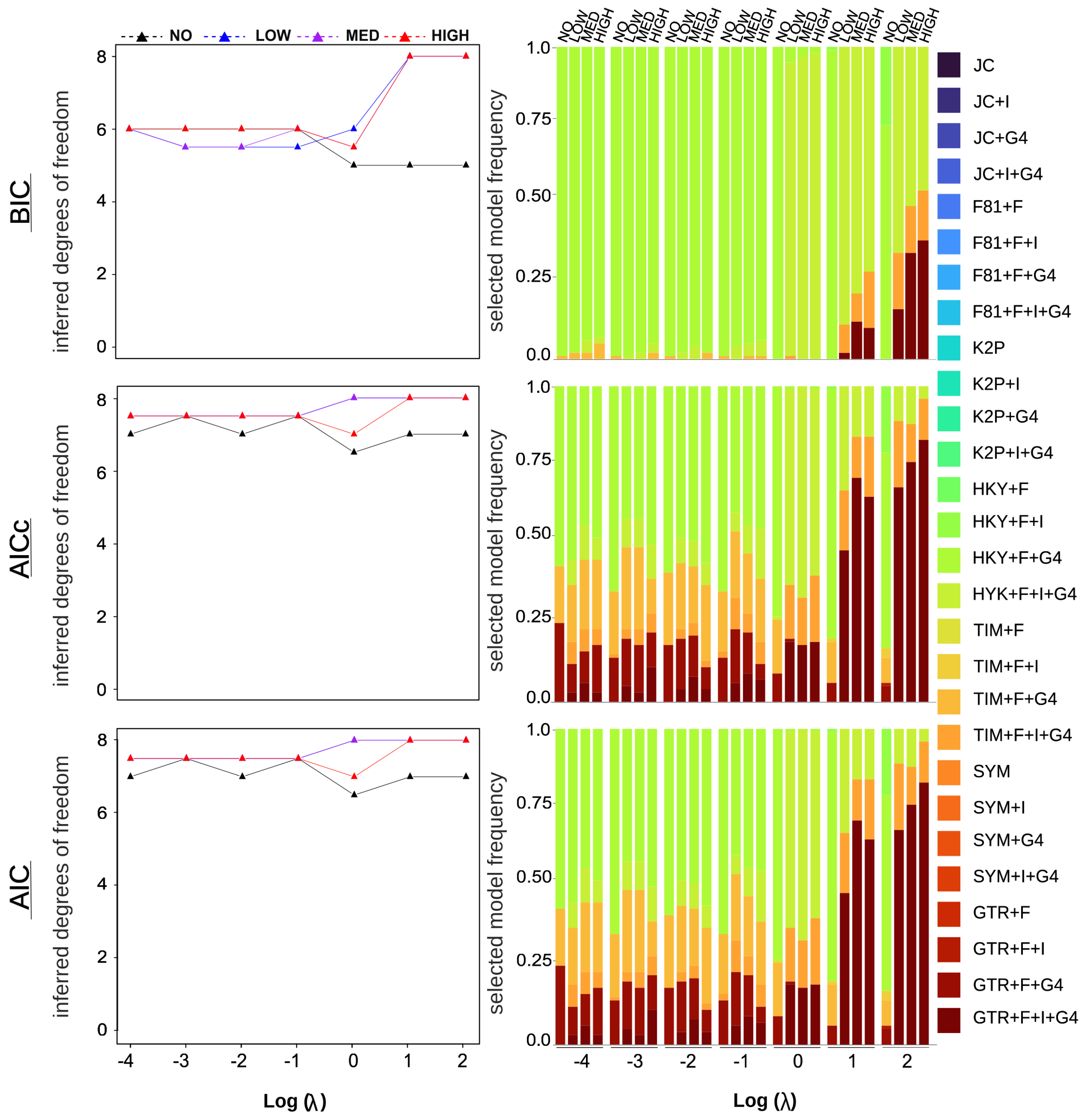

FIGURE S8. Results of nucleotide model selection across four levels of phylogenetic conflict and increasing speciation rates for analyses with $n = 100$ species. Panels in the left column show the average number of free parameters (degrees of freedom) inferred from model selection under each criterion (rows). Stacked bar plots in the right columns show the frequency fractions of the 28 substitution models selected for the conflict and speciation rate increase (left to right). In these supplementary analyses, all data were generated according to the HKY+G4 model.

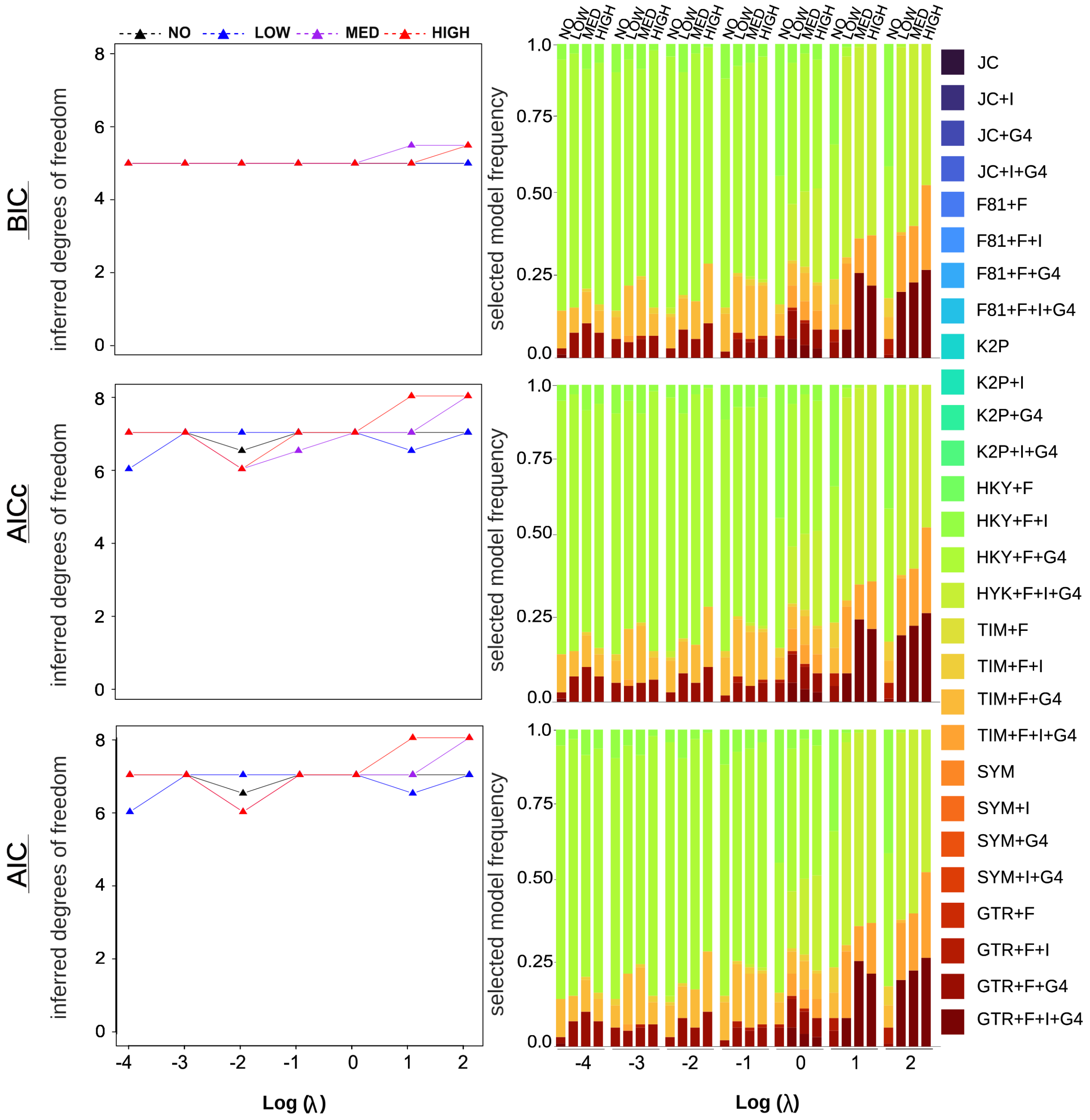

FIGURE S9. Results of nucleotide model selection across four levels of phylogenetic conflict and increasing speciation rates for analyses with $n = 10$ species. Panels in the left column show the average number of free parameters (degrees of freedom) inferred from model selection under each criterion (rows). Stacked bar plots in the right columns show the frequency fractions of the 28 substitution models selected for the conflict and speciation rate increase (left to right). In these supplementary analyses, all data were generated according to the HKY+G4 model.
